# Immune Evasion Mechanisms of *Entamoeba histolytica*: Progression to Disease

**DOI:** 10.3389/fmicb.2015.01394

**Published:** 2015-12-15

**Authors:** Sharmin Begum, Jeanie Quach, Kris Chadee

**Affiliations:** Department of Microbiology, Immunology and Infectious Diseases, Cumming School of Medicine, Snyder Institute for Chronic Diseases, University of CalgaryCalgary, AB, Canada

**Keywords:** *Entamoeba histolytica*, immune evasion, phagocytosis, apoptosis, trogocytosis

## Abstract

*Entamoeba histolytica* (*Eh*) is a protozoan parasite that infects 10% of the world's population and results in 100,000 deaths/year from amebic dysentery and/or liver abscess. In most cases, this extracellular parasite colonizes the colon by high affinity binding to MUC2 mucin without disease symptoms, whereas in some cases, *Eh* triggers an aggressive inflammatory response upon invasion of the colonic mucosa. The specific host-parasite factors critical for disease pathogenesis are still not well characterized. From the parasite, the signature events that lead to disease progression are cysteine protease cleavage of the C-terminus of MUC2 that dissolves the mucus layer followed by *Eh* binding and cytotoxicity of the mucosal epithelium. The host mounts an ineffective excessive host pro-inflammatory response following contact with host cells that causes tissue damage and participates in disease pathogenesis as *Eh* escapes host immune clearance by mechanisms that are not completely understood. Ameba can modulate or destroy effector immune cells by inducing neutrophil apoptosis and suppressing respiratory burst or nitric oxide (NO) production from macrophages. *Eh* adherence to the host cells also induce multiple cytotoxic effects that can promote cell death through phagocytosis, apoptosis or by trogocytosis (ingestion of living cells) that might play critical roles in immune evasion. This review focuses on the immune evasion mechanisms that *Eh* uses to survive and induce disease manifestation in the host.

## Introduction

*Entamoeba histolytica* (*Eh*), the causative agent of amebiasis, is an intestinal protozoan parasite that colonizes the intestinal lumen asymptomatically (known as noninvasive disease) in approximately 90% of cases. However, in 10% of individuals, this asymptomatic relationship breaks down and the parasite breaches the innate mucosal barrier and invades the underlying lamina propria resulting in 100,000 death per year globally (Stanley, 2003). Parasite cysts are acquired through the ingestion of contaminated food and water mostly in areas of poor sanitation (Ralston and Petri, 2011a). Birth cohort studies done in an urban slum in Dhaka, Bangladesh found that approximately 50% of infants are infected in the first year of life, with repeated infections that are connected with malnourishment and stunting (Mondal et al., 2006; Korpe et al., 2013). The exact reasons why *Eh* occasionally invade the mucosal epithelium and what host-parasite factors are involved in parasite invasion are not clear. The outcome of invasive *Eh* infection is variable; it can result in amebic diarrhea, amebic colitis, and/or dissemination of the parasites through the portal circulation to cause liver abscess (Ralston and Petri, 2011b). *Eh* invasion induces a robust pro-inflammatory response and host tissue destruction that exacerbates disease (Moonah et al., 2013; Mortimer et al., 2015). Currently, there is no effective vaccine for this disease; however nitroimidazoles (such as metronidazole) are effective to treat this tissue dwelling parasites. Metronidazole treatment can cause toxic side effects and enhance the probability of developing drug resistant (Petri, 2003; Ralston and Petri, 2011b). Our host immune system sets up a series of defensive responses against the parasite. However, continued morbidity and mortality point out that this parasite is capable of escaping host defense responses to maintain its own survival (Moonah et al., 2013). Thus, an understanding of the human immune response to the parasite and the strategies used by the parasite to evade host defense will deeply improve the development of effective immunotherapies. In this review, we will focus on the host immune responses against *Eh* and the complex strategies the parasite uses to evade host immunity.

## Host immunity against *E. histolytica*

### Innate and adaptive immune response

For any ingested parasite, immunity begins from the stomach acid as it has the ability to kill acid-sensitive microorganisms. However, the mucus barrier of the intestine serves as the first protective layer that prevents *Eh* from making contact with the underlying intestinal epithelial cells (IECs; Moonah et al., 2013). There are three major virulent factors of *Eh* known to mediate pathogenicity: (1) galactose/N-acetylgalactosamine-inhibitable lectin (Gal-lectin) is responsible for binding colonic mucin in colonization and host cell adhesion in disease pathogenesis, (2) pore forming peptide amoebapore used for host cell killing and, (3) cysteine proteases that lyse host extracellular matrix (Campos-Rodríguezp and Jarillo-Luna, 2005) and stimulate pro-inflammatory responses. The mucus barrier in the colon is composed of MUC2 mucin, which is a glycoprotein secreted from goblet cells. Mucin binds with high affinity to *Eh* Gal-lectin allowing the parasite to colonize the gut and at the same time it acts as a physical barrier to inhibit parasite invasion of the underlying epithelium (Chadee et al., 1987; Moonah et al., 2013). When *Eh* overcomes innate host defenses and contact IECs they produce a variety of pro-inflammatory mediators/chemokines including interleukin-1 (IL-1β), interleukin-8 (IL-8), and TNF-α. Several of these mediators trigger the recruitment of immune cells including neutrophils and macrophages to the site of parasite invasion (Yu and Chadee, 1997). The main amebicidal activity of neutrophils is the release of reactive oxygen species (ROS; Guerrant et al., 1981; Denis and Chadee, 1989). In macrophages *Eh* Gal-lectin up-regulates the mRNA expression of different cytokines. Gal-lectin induces pattern recognition receptor (PRRs) such as TLR-2 and TLR-4 mRNA expression in macrophages which is controlled by nuclear factor NF-κB and MAPK pathway. Recognition of parasite molecules by surface PRRs are crucial for the up-regulation of pro-inflammatory cytokine expression via NF-κB (Kammanadiminti et al., 2004). Macrophages activated with cytokines such as IFN-γ or TNF-α kills *Eh* trophozoites *in vitro* by producing nitric oxide (NO) from L-arginine mediated by inducible nitric oxide synthase (iNOS) (Lin et al., 1994). NO is critical for macrophage-mediated killing as iNOS deficient mice are more vulnerable to amebic liver abscess (ALA) and hepatocytic apoptosis (Seydel et al., 2000). Interferon gamma (IFN-γ) is the major cytokine that activates neutrophils and macrophages to exert amebicidal activity. Higher levels of IFN-γ are related to a lower incidence of *Eh* infection (Denis and Chadee, 1989; Ghadirian and Denis, 1992; Haque et al., 2007).

Humoral immune responses against *Eh* are well characterized and it was been found that 81–100% of invasive amebiasis patients develop circulatory antibodies within 7 days of infection (Kaur et al., 2004). A prospective cohort study of pre-school children in Dhaka, Bangladesh, showed that mucosal IgA antibodies against the carbohydrate recognition domain (CRD) of the Gal-lectin heavy chain provided protection against *Eh* infection and disease (Haque et al., 2001, 2006). In contrast, serum anti-lectin IgG was not associated with protection but mainly with the frequency of new infection (Haque et al., 2001). Higher levels of anti-lectin IgG was found in ALA and intestinal amebiasis as compared to asymptomatic patients (Kaur et al., 2004). These findings indicate that systematic anti-lectin antibodies are not involved in direct protection against amebiasis.

### Inflammasome activation by *E. histolytica*

*Eh* imposes damage through the adherence to host cells, which plays a critical role in killing or ingesting host target (Mortimer and Chadee, 2010). Thus, amebae adherence to host cell is one of the major characteristics of *Eh* pathogenicity. At present, the pattern recognition receptors (PRRs) that bind *Eh* Gal-lectin are not known. We recently identified that the inflammasome pathway is only activated on contact with live *Eh* and distinguishes between different physical forms of *Eh* (Mortimer et al., 2014). Inflammasome is a cytosolic multiprotein complex, which acts as a sensor for pathogens and cellular damage. This multimeric complex consists of an inflammasome sensor molecule (NOD-like receptor), the adaptor protein ASC and caspase-1. Activation of inflammasome leads to rapid and robust secretion of IL-1β, IL-18, IL-1α, FGF-2, IP-10 (Mortimer et al., 2014). Interestingly, when *Eh* activates the inflammasome, it does not trigger caspase-1 dependent cell death (known as pyroptosis) (Mortimer et al., 2014). It is unclear if inflammasome-activated macrophages are amebicidal and whether it plays other protective roles in amebic infection. On the other hand, if inflammasome activation triggers cell death in macrophages (pyroptotic cell death), *Eh* can use it as an advantage to limit immune elimination that can become detrimental to host defense.

## Immune evasion mechanisms of *E. histolytica*

*Eh* has a two-phase life cycle: it can survive as an infective cyst in the environment or it can be found as trophozoites, the feeding and tissue dwelling stage in the human colon. After excystation in the colon, *Eh* trophozoites usually establishes harmless colonization where the parasites reside in the gut lumen and feed on enteric bacteria by phagocytosis (Voigt et al., 1999; Wilson et al., 2012). However, for unknown reasons trophozoites can become invasive, where parasite virulence factors allow it to degrade colonic mucin and other innate epithelial barrier functions (Wilson et al., 2012). Host immune responses, both innate and adaptive, are robust against invasive *Eh* but still this parasite is able to survive by developing immune evasion strategies. In particular, *Eh* cysteine proteases can cleave MUC2 mucin abrogating its protective functions allowing the parasite to breach the mucus layer and attach to the underlying epithelial cells (Lidell et al., 2006). Intestinal antimicrobial peptides are also an important component of host innate immune defense. Even though human LL-37 and murine CRAMP (cathelin-related antimicrobial peptide) cathelicidins are induced by *Eh* trophozoites both at the mRNA and protein level in IECs, *Eh* cysteine proteases can cleave these antimicrobial peptides (Cobo et al., 2012). Thus, *Eh* is resistant to both intact and cleaved antimicrobial cathelicidins in the intestine (Cobo et al., 2012).

After amebic invasion, neutrophils are the earliest infiltrating cells but virulent *Eh* are effective in killing, lysing, and phagocytosing neutrophils. *In vitro*, one trophozoite was shown to kill approximately 3000 neutrophils (Guerrant et al., 1981; Guo et al., 2007). There are several conflicting mechanisms by which ameba interfere with neutrophil functions. *Eh* can disrupt NADPH oxidase activities and inhibit the respiratory burst of neutrophils to avoid oxidative stress. *Eh* iron-containing superoxide dismutase and NADPH:flavin oxidoreductase (Elnekave et al., 2003) are able to detoxify ROS by forming H_2_O_2_ (Bruchhaus et al., 1998; Sim et al., 2005). *Eh* trophozoites can protect themselves from neutrophil reactive oxygen properties with a 29-kDa surface protein, peroxiredoxin that has potent antioxidant activity (Davis et al., 2006). Studies have shown (Sim et al., 2005) that *Eh* can induce host cells (neutrophil) apoptosis through the activation of ERK1/2 by the generation of NADPH oxidase-derived ROS.

Macrophages, another effector cell present during amebic infection also show suppressed cell mediated immunity due to *Eh*-induced strategic immune modulation. *Eh* trophozoites inhibit respiratory burst (ROS: H_2_O_2_, O^2−^, OH^−^) and NO production by macrophages (Lin et al., 1993; Wang et al., 1994). NOS substrate L-arginine is competitively converted to L-ornithine by ameba arginase that limit NO production by macrophages (Elnekave et al., 2003). When *Eh* and macrophages are exposed to each other, ameba produces the immunoregulatory molecule prostaglandin E2 (PGE_2_), synthesized by a cyclooxygenase (COX)-like enzyme by the parasite (Dey et al., 2003). Coupling through EP2/4 receptors, PGE_2_ increases cyclic adenosine monophosphate (cAMP) levels in macrophages that inhibits Th1 cytokine release, NADPH-mediated oxidative burst, and NO synthesis through the protein kinase C (PKC) pathway (Wang and Chadee, 1995). Another immunosuppressive pentapeptide, monocyte locomotion inhibitory factor (MLIF) produced by *Eh* showed anti-inflammatory activities by inhibiting NO production (Rico et al., 2003).

The complement system of the host is able to prevent trophozoite dissemination into the extra intestinal space. Activated complement forms the membrane attack complex (MAC) that can potentially lyse the parasite. *Eh* resists complement activation by the Gal-lectin which have sequence resemblance and antigenic cross reactivity with the MAC-inhibitory protein CD59 and thus inhibit MAC-mediated lysis (Braga et al., 1992). Cysteine proteases can also cleave complement components (Reed et al., 1995). The potent pro-inflammatory activities of the complement component C3a and C5a are degraded by *Eh* secreted extracellular cysteine proteases (Zambrano-Villa et al., 2002). Secretory IgA and serum IgG mediate adaptive immunity against *Eh* and ameba can degrade these immunoglobulins *in vitro*. *Eh* extracellular cysteine proteases play a key role in the disruption of host adaptive defenses. For successful invasion, *Eh* secreted and membrane-bound cysteine proteases cleave extracellular matrix proteins, fibronectin, and laminin and avoid host defenses by cleaving gut lumen sIgA and circulatory IgG (Que and Reed, 1997; Zambrano-Villa et al., 2002).

### *E. histolytica*-induced cell death: Immune evasion strategy

*Eh* uses different strategies to evade host immune defense but one striking mechanism is the induction of host cell death. The term “histolytica” refers to *Eh* ability to destroy host tissues by potent cytotoxicity/cell killing activity toward different host cells including neutrophils, macrophages, T-lymphocytes; though the exact mechanism of host cell killing is not clear. *Eh* has several cytotoxic effector molecules (Table 1) but how ameba deliver this deadly action is not clear. *Eh* can induce host cell apoptosis, phagocytosis and amebic trogocytosis; the latter involving a recently described mechanism of ameba-induced host cell killing (Ralston et al., 2014).

**Table 1:** **Potential cytotoxic effector molecules identified in ***Entamoeba histolytica*****.

***Eh* effectors molecules**	**Identified effect on host cell**	**References**
Amebapores	Pore forming proteins	Leippe et al., 1991
	All three types induce pore formation in synthetic liposomes	Andrä et al., 2003
Amebapore A	Active at low pH 5.2. May play a role in host cell killing before ingestion	Andrä et al., 2003
Amebapore B Amebapore C	*In vitro*, purified amebapores showed bactericidal activity against Gram-positive bacteria at nanomolar concentration Purified amebapores are cytotoxic to Jurkat or U937 cells at micromolar concentration	Leippe et al., 1994; Andrä et al., 2003
Cysteine proteinases (CPs)	At least 50 CP genes are encoded and some of them are secretory	Tillack et al., 2007
	Proteinases act on a variety of host substrates such as mucin, villin, laminin, collagen, proteoglycan, and extracellular matrix (ECM). It plays a role in pathogenesis by cleaving MUC mucin and ECM degradation	Li et al., 1995; Lidell et al., 2006
	*In vivo*, overexpression of *Eh*CP5 (*Eh*CP5) increases liver abscess formation compared to wild-type controls. Other CPs like *Eh*CP1 or *Eh*CP2 overexpression had no effect	Hellberg et al., 2001; Tillack et al., 2006
Membrane proteins: Gal/GalNAc lectin associated	35kDa light subunit of the Gal/GalNAc lectin and surface-localized thiol-dependent peroxidase	Ankri et al., 1999; Sen et al., 2007
	Antisense inhibition of both of these prevents cell killing and deceased liver abscess	Ankri et al., 1999; Sen et al., 2007
*Eh*STIRPs (*Eh* serine, threonine and isoleucine rice proteins)	Silenced by dsRNA resulted in defects in both adhesion and cytotoxicity	MacFarlane and Singh, 2007
*Eh*TMKB1-9 (*Eh* transmembrane kinase B1-9)	Antisense inhibition of this protein showed defects in both adhesion and host cell killing	Shrimal et al., 2010
KERP1 (Lysine and glutamic acid rice protein 1)	Parasite membrane protein binds to host cell membrane	Santi-Rocca et al., 2008
	Antisense inhibition of this protein failed to decrease mRNA but due to affinity for host cell membranes, it might have role in cytotoxicity	

#### Apoptotic cell death

**Figure 1:**
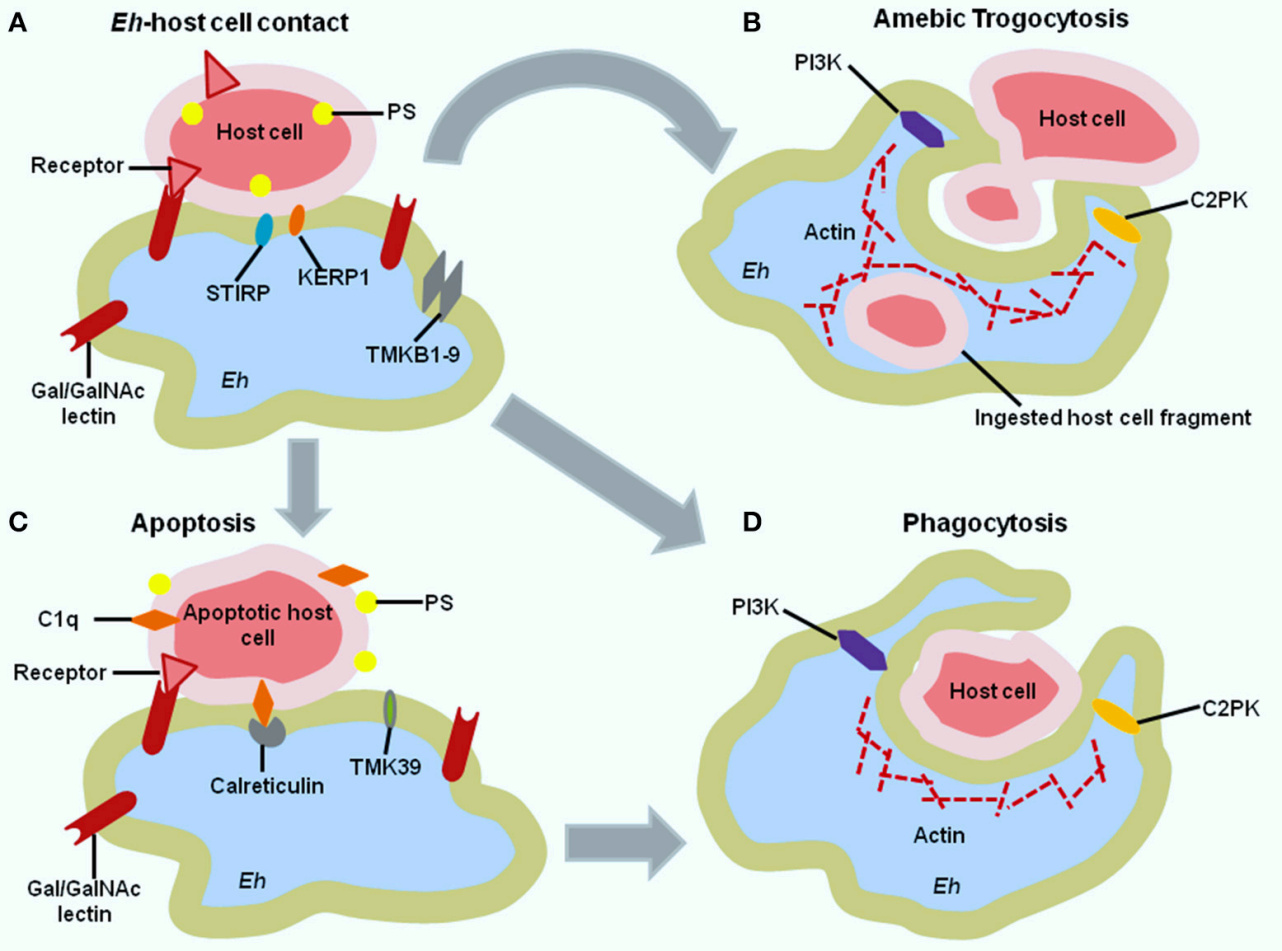
**Immune evasion of ***E. histolytica*** (***Eh***) by host cell killing. (A)** Contact between *Eh* and host cell is mediated by the Gal-lectin and host cell surface Gal and GalNAc receptors. Other amebic proteins involved in host cell attachment include the trans-membrane serine, threonine, and isoleucine proteins (STIRP) and transmembrane kinase family member TMKb1-9. After *Eh*-host contact, three events can take place-**(B)** amebic trogocytosis **(C)** apoptotic cell death **(D)** phagocytosis. In **(B**) larger cells undergo amebic trogocytosis. Amebic trogocytosis require PI3K and C2PK signal transduction for actin polymerization. **(C)** Host cells that have been induced to undergo apoptosis express phosphatidylserine (PS) and C1q complement protein that induce opsonization by ameba involving amebic calreticulin receptor. Finally, in **(D)** both apoptotically death cell and smaller cell undergo phagocytosis. Signal transduction for phagocytosis is also mediated by PI3K and EhC2PK and influences actin polymerization. PI3K-phosphoinositide 3 kinase, C2PK-C2 domain containing protein kinase, TMK39-Transmembrane kinase family member.

Host cell killing is usually a stepwise process mediated by parasite adherence to the target cell, elevation of intracellular calcium level, dephosphorylation of host proteins which all contribute to cell death via activation of caspase-3 (Ralston and Petri, 2011b). Caspase-3 activation is the signature event of apoptosis. The first step of adherence is mediated by the parasite surface Gal-lectin to host cell carbohydrate determinants containing Gal and/or GalNAc residues (Figure 1A). Gal-lectin mediated adherence to target cell is a prerequisite for parasite cytotoxicity as the addition of excess Gal or GalNAc monomers inhibit *Eh* adherence and target cell killing (Ravdin and Guerrant, 1981; Saffer and Petri, 1991). Adherence to the target cell induces calcium flux which also contribute to the cell killing proved by the inhibition of calcium channel or by using calcium chelators (Ravdin et al., 1982; Ralston and Petri, 2011b). Amebic cytotoxicity both *in vitro* and *in vivo* occurs via the caspase-3 dependent apoptotic cell death pathway in Jurkat cells after contact with *Eh* (Figures 1A,C) (Seydel and Stanley, 1998; Ralston and Petri, 2011b). Caspase-3 knockout mice are resistant to amebiasis and a pharmacological inhibitor of caspase-3, Ac-DEVD-CHO (N-acetyl-Asp-Glu-Val-Asp-aldehyde) reduces parasite cytotoxicity to host cells (Huston et al., 2000; Becker et al., 2010). Studies illustrate that *Eh*-induced apoptosis is independent of caspase-8 or caspase-9 (Huston et al., 2000). Another study showed that *Eh* killing of hepatocyte and immune cell was not dependent or mediated by the classical Fas/Fas ligand or TNFα receptor pathway (Seydel and Stanley, 1998). Mice lacking Fas or producing a mutated non-functional Fas protein developed amebic ALA similar to wild type mice. Similarly, TNF receptor knockout mice and wild type mice develop comparable ALA (Seydel and Stanley, 1998). *Eh* induces a non-classical pathway of apoptosis that may have an important role in pathogenesis (Seydel and Stanley, 1998; Huston et al., 2000). Apoptotic cell death is immunologically silent, thus induction of host cell apoptosis by *Eh* tricks the host cell to kill itself without evoking an inflammatory response to avoid being detected by other immune cells. The importance of apoptosis in regards to amebic virulence is brought to light by studies of the leptin-signaling pathway. The hormone leptin is linked to malnutrition (signals satiety) and regulates the immune response to infection through the Th1 inflammatory response and by preventing apoptosis (Wilson et al., 2012). Leptin signaling provides protection from mucosal destruction and experiments in mice showed the anti-apoptotic role of leptin in gut epithelia (Guo et al., 2011). Polymorphism (even a single amino acid substitution) in the leptin receptor was found to be associated with increased *Eh* infection susceptibility (Duggal et al., 2011).

#### Phagocytosis by *E. histolytica*

The fate of *Eh* induced apoptotic host cells (Huston et al., 2003) and subsequent phagocytosis may play an important role in the host-parasite relationship in amebiasis. Amebae induced phagocytosis of erythrocyte is one of the possible distinctive feature of *Eh* from the commensal *E. dispar* (González-Ruiz et al., 1994). In multicellular organisms, phagocytosis is the last step of the apoptotic pathway to clear dead cells without provoking an inflammatory response by the toxic components of dead cells (Figure 1D; Savill and Fadok, 2000; Fadok et al., 2001). Similarly, apoptotic host cells are phagocytosed by *Eh* with the help of exposed phosphatidylserine (Huston et al., 2003). As *Eh* rapidly clears apoptotically killed host cells by phagocytosis, this limits the spillage of toxic intracellular contents from killed cells. Through this strategy *Eh* restrains host inflammatory responses and build up prolonged infection.

*Eh*-induced phagocytosis is important for pathogenicity but very little is known about the amebic receptors and the corresponding ligands that they bind to. Adherence with host cells by parasite Gal-lectin is critical for cell death but blocking of adherence does not prevent phagocytosis (Teixeira et al., 2008). This suggests the sequential exposure of new ligands on dying cells as well as the recruitment of new receptors on *Eh* in addition to the Gal-lectin following host cell killing (Teixeira et al., 2008). From an evolutionary concept, *Eh* phagocytoses bacteria for nutrient acquisition; therefore amebae preferentially recognize and phagocytose apoptotic cells that have surface similarities with bacteria (Teixeira et al., 2008).

#### Ameba trogocytosis

Recently, a new mechanism of *Eh* killing came to light. Using live cell imaging technology it was discovered that following host cell attachment, *Eh* trophozoites ingest separate parts (bites) of host cells which was termed “amebic trogocytosis” (Figure 1B; Ralston et al., 2014). This was a very rapid process as within 1 min of attachment amebic trogocytosis is initiated. Due to biting off and ingestion of separate pieces of host cells, intracellular calcium levels were elevated and this triggered ultimate cell death, evidenced by the loss of cell membrane integrity (Ralston et al., 2014). Interestingly, when trophozoites were incubated with either live or pre-killed host cells, only live cells were seen to be trogocytosed by ameba and pre-killed cells were ingested whole (phagocytosed; Ralston et al., 2014; Ralston, 2015). The cell surface characteristics of pre-killed host cells might be different from the directly killed cells and *Eh* might use this surface difference to determine the type of ingestion (Ralston, 2015). After ingestion of bites, amebae detach from the host cell, and the ingested cell eventually dies. It was speculated that this process contributes to amebic invasion in the colon as *Eh* can also trogocytose mucosal epithelial cells. Though amebic trogocytosis is a rapid process, this depends on specific conditions like physiological temperature, amoebic actin rearrangements, Gal-lectin, EhC2PK (*Eh* C2-domain-containing protein kinase), and PI3K (phosphoinositide 3-kinase) signaling (Ralston et al., 2014). Interference with any of these protein was shown to reduce *Eh* trogocytosis and subsequent decrease in host cell death (Ralston, 2015). It should be noted that trogocytosis also occurs in multicellular organism and different immune cell types but this trogocytosis does not trigger cell death. The exact reason for this distinction is not clear but trogocytosis in multicellular organisms mainly involves the exchange of cell membrane fragments; whereas *Eh* trogocytosis contains target cell cytoplasm and sometimes organelles (Joly and Hudrisier, 2003; Ralston et al., 2014).

The concept of trogocytosis raises an important question, whether amebic trogocytosis is different from phagocytosis. The signaling proteins involved in amebic trogocytosis also play important roles during *Eh* phagocytosis but amebic trogocytosis is predominant in living cells (Ralston, 2015). Some factors might take part in the distinction of trogocytosis and phagocytosis like target cell deformability, target cell viability, target cell size etc. (Ralston, 2015). However, the specific signaling pathways that define amebic trogocytosis and phagocytosis in *Eh* are not identified; the relationship or differences between these two processes are not well understood.

## Conclusion

*Eh* is an enteric dwelling protozoan parasite that causes significant morbidity and mortality in developing countries. This parasite can develop a harmless colonization in the colon and for unknown reasons it can become a pathogenic phenotype. With the pathogenic phenotype, *Eh* disrupts innate mucosal barriers and penetrates the underlying lamina propria where the parasite develops potent cytotoxic activity and extensive tissue destruction. It is still not clear how or what factors induce this pathogenic phenotype. Human and parasite genetics along with environmental factors might have a role as it has been found that not all children are equally susceptible to infection. Malnutrition extensively increases disease susceptibility. Gut microbiome also influence *Eh* infection susceptibility. Both host innate and adaptive immune response take part in the elimination of invasive *Eh*. The host immune system builds up a rapid inflammatory response by the secretion of cytokines/chemokines, recruitment of immune cells (neutrophils, macrophages), and the activation of inflammasome to control invasive parasites. This parasite also develops multiple strategies to subvert host immune responses and to promote its own survival. *Eh* induces host cell killing primarily by apoptosis, which is a non-inflammatory cell death mechanism. Induction of apoptotic cell death is an active and stepwise process. After inducing cell death, the parasite also clears the corpse by phagocytosis to inhibit further inflammatory responses. Another mechanism is amebic trogocytosis where *Eh* bites live cells very rapidly and induces cell death. The proper characterization of proteins, receptor/ligand interaction involved in parasite adherence, cell killing, phagocytosis, and amebic trogocytosis will provide promise of future vaccine candidates.

## Author contributions

SB, JQ, and KC wrote and edit the manuscript.

## Funding

This work was supported by a grant from the Natural Sciences and Engineering Research Council of Canada (NSERC). SB and JQ is supported by scholarships from NSERC CREATE.

### Conflict of interest statement

The authors declare that the research was conducted in the absence of any commercial or financial relationships that could be construed as a potential conflict of interest.
